# Visual Impairment, Eye Conditions, and Diagnoses of Neurodegeneration and Dementia

**DOI:** 10.1001/jamanetworkopen.2024.24539

**Published:** 2024-07-30

**Authors:** Erin L. Ferguson, Mary Thoma, Peter T. Buto, Jingxuan Wang, M. Maria Glymour, Thomas J. Hoffmann, Hélène Choquet, Shea J. Andrews, Kristine Yaffe, Kaitlin Casaletto, Willa D. Brenowitz

**Affiliations:** 1 Department of Epidemiology and Biostatistics, University of California, San Francisco; 2 Department of Epidemiology, Boston University, Boston, Massachusetts; 3 Kaiser Permanente Northern California, Division of Research, Oakland; 4 Department of Psychiatry and Behavioral Sciences, University of California, San Francisco; 5 Memory and Aging Center, Department of Neurology, Weill Institute for Neurosciences, University of California, San Francisco; 6 Kaiser Permanente Center for Health Research, Portland, Oregon

## Abstract

**Importance:**

Vision and eye conditions are associated with increased risk for Alzheimer disease and related dementias (ADRDs), but the nature of the association and the underlying biological pathways remain unclear. If causal, vision would be an important modifiable risk factor with viable population-level interventions.

**Objective:**

To evaluate potentially causal associations between visual acuity, eye conditions (specifically cataracts and myopia), neuroimaging outcomes, and ADRDs.

**Design, Setting, and Participants:**

A cohort and 2-sample bidirectional mendelian randomization (MR) study was conducted using UK Biobank participants and summary statistics from previously published genome-wide association studies on cataract, myopia, and AD. The participants included in the analysis were aged 55 to 70 years without dementia at baseline (calendar years 2006 to 2010), underwent genotyping, and reported on eye conditions; a subset completed visual acuity examinations (n = 69 852-71 429) or brain imaging (n = 36 591-36 855). Data were analyzed from August 15, 2022, through November 28, 2023.

**Exposure:**

Self-reported cataracts, visual acuity, and myopia measured by refraction error.

**Main Outcomes and Measures:**

ADRD, AD, and vascular dementia were identified from electronic medical records. Total and regional brain volumes were determined using magnetic resonance imaging.

**Results:**

The sample included 304 953 participants (mean [SD] age, 62.1 (4.1) years; 163 825 women [53.72%]); 14 295 (4.69%) had cataracts and 2754 (3.86%) had worse than 20/40 vision. Cataracts (hazard ratio [HR], 1.18; 95% CI, 1.07-1.29) and myopia (HR, 1.35; 95% CI, 1.06-1.70) were associated with a higher hazard of ADRD. In MR analyses to estimate potential causal effects, cataracts were associated with increased risk of vascular dementia (inverse variance-weighted odds ratio [OR], 1.92; 95% CI, 1.26-2.92) but were not associated with increased dementia (OR, 1.21; 95% CI, 0.98-1.50). There were no associations between myopia and dementia. In MR for potential reverse causality, AD was not associated with cataracts (inverse variance–weighted OR, 0.99; 95% CI, 0.96-1.01). Genetic risk for cataracts was associated with smaller total brain (β = −597.43 mm^3^; 95% CI, −1077.87 to −117.00 mm^3^) and gray matter (β = −375.17 mm^3^; 95% CI, −680.10 to −70.24 mm^3^) volumes, but not other brain regions.

**Conclusions and Relevance:**

In this cohort and MR study of UK Biobank participants, cataracts were associated with increased risk of dementia, especially vascular dementia, and reduced total brain volumes. These findings lend further support to the hypothesis that cataract extraction may reduce the risk for dementia.

## Key Points

**Question:**

Are eye conditions and visual acuity risk factors for Alzheimer disease (AD) and related dementias?

**Findings:**

In this cohort study of 304 953 UK Biobank participants, cataracts were associated with lower total gray matter volume, white matter hyperintensities, and increased risk of dementia, especially vascular dementia. Mendelian randomization analyses estimated that cataracts were associated with a 92% increase in the odds of vascular dementia risk; although poor visual acuity was associated with an increased risk of dementia, myopia was not associated with dementia in genetic analyses.

**Meaning:**

These results suggest that cataracts increase the risk of dementia through vascular and non-AD mechanisms; treating or preventing cataracts may reduce dementia risk.

## Teaser

This cohort study examines visual acuity and eye conditions in individuals with genetic risk for Alzheimer disease and related dementias.

## Introduction

Visual impairment and ocular disease are emerging as potential modifiable risk factors for Alzheimer disease (AD) and related dementias (ADRDs).^1^ Self-reported vision impairment,^2,3^ eye conditions such as cataracts,^4,5^ and poor visual acuity^6,7,8^ are associated with an increased risk of dementia. If causal, this association would be clinically meaningful; some vision impairments can be reversed or corrected. For example, observational studies have reported that cataract surgery is associated with a decreased risk of dementia.^4,9^

Establishing the causality of this association is methodologically difficult. Both vision impairment and ADRDs can develop slowly over decades, they share risk factors,^10,11^ and early ADRDs could affect visual processing (reverse causation).^12^ Additionally, previous studies are observational and may be affected by unmeasured confounding by social or other health factors^13,14,15^ or confounding by indication.^16^ Alternative approaches are needed to inform whether vision care could delay the onset of ADRD.

Genetic variants associated with eye conditions or AD^17,18,19^ can be leveraged to estimate causal associations, because genetic variants are likely independent of key confounders. Mendelian randomization (MR) uses genetic variants, single-nucleotide variants (SNVs), as instruments, allowing for an unbiased estimate of causal effect, provided certain assumptions are met.^20,21^ Few studies have used MR methods to evaluate associations between vision impairments and dementia. One study reported null findings for the association between cataracts and AD; however, this study did not examine vision impairments or dementia more broadly.^22^ Furthermore, previous studies have not investigated related indicators of ADRD, such as neuroimaging markers, which would provide convergent evidence and elucidate biological pathways.

**Figure 1.**
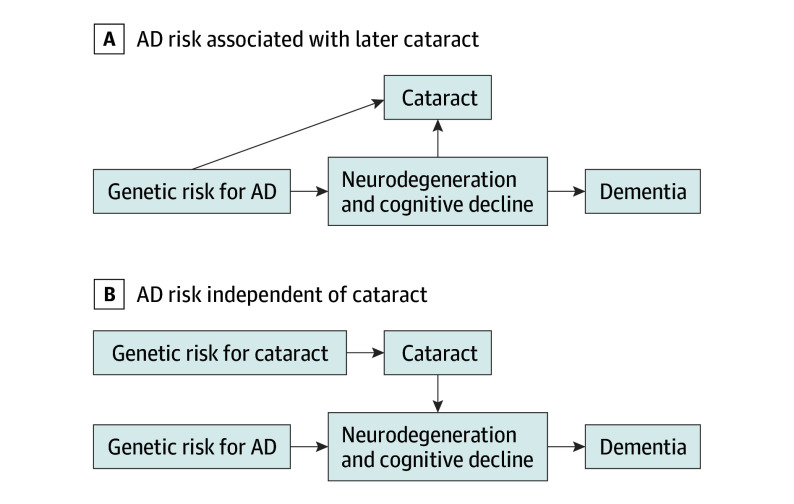
Hypothesized Associations Between Cataracts or Other Eye Conditions and Brain Regions, Leading to Dementia With mendelian randomization, variations in genetic risk can be used to distinguish between 2 causal scenarios. A, If incipient neurodegeneration or other physiological markers cause cataracts to form, then genetic risk for Alzheimer disease (AD) should be associated with later cataracts. B, If cataracts have independent effects on processes leading to dementia (ie, cataracts are a causal risk factor for dementia), then genetic risk for AD should be independent of cataracts and that genetic risk for cataracts would be associated with dementia.

We evaluated the possible associations between visual impairment, cataracts, and ADRD in the UK Biobank (UKB). With recent interest in visual acuity^23^ and cataracts^9^ as dementia risk factors, we focused our genetic analyses on cataracts and myopia. With MR, we first used genetic risk for AD to evaluate whether shared genetics or incipient AD increases the risk of later vision impairment or eye conditions (Figure 1A). Second, we used genetic risk for eye conditions (cataracts and myopia) to evaluate whether incipient eye conditions increase the risk of ADRD, vascular dementia (VaD), or AD (Figure 1B). Third, to explore biological pathways, we evaluated eye conditions and brain magnetic resonance imaging (MRI) outcomes.

## Methods

### Study Population

The UKB is an ongoing biobank study that enrolled more than 500 000 individuals aged 40 to 69 years from 2006 to 2010 across the UK.^24^ Participants provided blood samples, survey responses, and their electronic health records are linked for follow-up through November 2021; a subset completed visual acuity tests (calendar years 2006-2010) and brain MRI (calendar years 2014-2010). This cohort study followed the Strengthening the Reporting of Observational Studies in Epidemiology using Mendelian Randomization (STROBE-MR) guidelines (eAppendix 1).^25^ Ethics approval for data collection was obtained from the National Health Service National Research Ethics Service, and all participants provided written informed consent. Participants receive compensation for travel.

We included participants aged 55 years or older in this analysis (age 55-70 years at baseline). We excluded individuals with dementia at baseline or without self-reported visual history. Analyses using genetic risk score (GRS) or MR were further restricted to those with European genetic ancestry due to concerns that variants may not generalize to those with non-European ancestry.

### Eye Disorders and Visual Acuity

Participants self-reported their history of eye problems (cataracts, glaucoma, age-related macular degeneration, and diabetic retinopathy) during their baseline visit. Participants also self-reported their history of cataract surgery. For analyses in which cataracts were the outcome, we used additional diagnoses of senile cataracts that were obtained from linked electronic health record data through the end of 2021. In a subset, the presence of myopia was determined from refraction error, measured by noncycloplegic autorefraction,^26,27^ and visual acuity was measured in both eyes as the logMAR chart.^27^ The measure from the better eye was used. Binary 20/40 vision was calculated using a logMAR cutoff of 0.3.

### Dementia

Incident dementia cases, including AD, VaD, and nonspecific dementia, were ascertained based on dementia diagnoses from linked primary care and inpatient electronic health records through 2021 using *International Statistical Classification of Diseases and Related Health Problems, 10th Revision*, codes (eTable 1 in Supplement 1).^28,29^ In sensitivity analyses, we restricted the outcome to dementia subtypes (AD or VaD).

### Brain Volumes

We selected 5 neuroimaging regions of interest as secondary outcomes. Based on research showing their association with cognitive decline, we included measures of total brain volume,^30^ total gray matter volume,^31,32^ hippocampal volume (both hemispheres),^31,33^ white matter hyperintensity volume,^34^ and mean cortical thickness in an AD signature region.^35^ The AD signature region is a surface area–weighted average of cortical thicknesses across 6 regions. We also included the volume of 1 visual region (ie, lateral occipital cortex) under the hypothesis that visual impairment may cause atrophy in visual tracts and regions.^36,37^

Magnetic resonance imaging data were obtained from the first imaging visit and were acquired using identical scanners and the same protocol across sites.^38^ Volumetric, cortical thickness, and surface area measures were obtained using FreeSurfer. All volumetric measures were adjusted for intracranial volume.

### Genetic Instruments for Cataracts, Myopia, and AD

Samples were genotyped in batches using 2 closely related arrays.^39,40^ We selected genetic instruments based on previously published genome-wide association studies (GWAS) for cataracts,^18^ myopia,^19^ and AD.^17^ We created GRS based on genome-wide significant SNVs identified in each GWAS (*P* < 1 × 10^−8^ unless otherwise specified). Genetic risk scores were calculated by multiplying each individual’s risk allele count for each locus by the effect estimates for that SNV from the respective GWAS and summing scores to create a weighted sum.^41^ SNPs in linkage disequilibrium were removed (*r*^2^ > 0.3). A primary GRS for cataracts (cataracts-GRS) was calculated from 43 SNVs from a 23andMe replication GWAS (347 209 cases and 2 887 246 controls) on cataracts (eTable 2 in Supplement 1).^18^ We examined a secondary GRS for cataracts based on variants independent from those identified in the UKB to minimize any weak instrument bias.^42^ The secondary cataract-GRS was based on 7 SNVs (*P* < 1 × 10^−7^) identified from participants with European ancestry from the Genetic Epidemiology Research in Adult Health and Aging (GERA) study (28 092 cases and 50 487 controls). Final GRS were transformed into a *z* score. The primary GRS for myopia (myopia-GRS) was limited to 55 SNVs that replicated in 23andMe and were statistically significant (*P* < 1 × 10^−8^) from the 23andMe replication study (eTable 3 in Supplement 1). Secondary analyses used a myopia-GRS (106 SNVs) created from the study’s GERA subcohort. In addition, a GRS for AD (AD-GRS) was created using effect estimates from a large GWAS on dementia that did not include the UKB.^17^ We focused on genetic risk for AD since genetic risk for AD is well described. The final AD-GRS was a weighted sum of 27 SNVs, including 2 apolipoprotein E (*APOE*) ε4 alleles (eTable 4 in Supplement 1). We replicated our analyses using a version of the AD-GRS without the *APOE* alleles.

### Covariates

Age at visit (baseline or imaging), self-reported sex (male and female), self-reported racial and ethnic identity (Asian, Black, Chinese, White, multiple races, and other), quintiles of index of multiple deprivation by country of origin (highest, above average, average, below average, and lowest), and 6 indicators for a history of relevant comorbidities (falls, broken bones, cardiovascular disease, stroke, diabetes, and problems hearing) were reported at baseline. We adjusted for racial and ethnic identity because there are racial disparities in visual impairment and dementia that we do not believe are explained by other covariates included in UKB data.^60,61^ Genetic models (including GRS and MR models) were adjusted for age, sex, and the first 10 principal components to adjust for confounding by population stratification. Imaging models included adjustment for imaging center. Models including visual acuity and myopia were additionally adjusted for use of corrective lenses.

### Statistical Analysis

#### Observational Analyses

We assessed the association between eye problems (cataracts, myopia, binary 20/40 vision, glaucoma, diabetic retinopathy, and age-related macular degeneration) and all-cause dementia, AD, and VaD using Cox proportional hazards regression models censored for death, loss to follow-up, or end of study period. In a secondary model focused on cataracts, we evaluated whether a history of cataract surgery modified associations with dementia risk. We also investigated cross-sectional differences in imaging markers using linear regressions.

#### Mendelian Randomization

To better investigate possible causality, we used genetic risk to evaluate the associations between cataracts, myopia, and dementia. There are 4 main assumptions that need to be met for MR.^43^ First, the relevance condition requires that SNVs are associated with the risk factor or exposure (eg, SNVs in the AD-GRS must be associated with AD). We confirmed that each GRS (cataracts-GRS, myopia-GRS, and AD-GRS) was associated with its corresponding exposure (eTable 5 in Supplement 1).^43^ Second, the independence assumption requires that there are no unmeasured confounders of the SNVs and outcome. Third, the exclusion restriction assumption states that the only effect of the SNVs on the outcome operates through the exposure. Fourth, we must assume monotonicity, meaning that our defined GRS does not have opposite effects on individuals within the sample.^44^

We first modeled the association of 2 cataracts-GRS and myopia-GRS with all-cause dementia using logistic regressions. To evaluate potential reverse causation, we additionally modeled the association between AD-GRS and 3 vision outcomes (cataracts, myopia, and binary 20/40 vision) using logistic regressions. These GRS allow us to leverage individual-level outcome data to examine potential associations. Next, we used 2-sample MR estimators and conducted sensitivity analyses to evaluate MR assumptions. Using the SNVs from each GRS as instruments, we conducted 2-sample MR assessing 5 associations: from (1) cataracts to all-cause dementia, (2) cataracts to VaD, (3) AD to cataracts, (4) AD to myopia, and (5) AD to 20/40 vision. Single-nucleotide variant associations with outcomes were estimated in the UKB using the same covariate adjustments (age, sex, and principal components) as the external GWAS for exposure data, and reference alleles were harmonized. We report estimates from 4 MR approaches^20^: MR Egger, weighted median, inverse variance weighted, and weighted mode. Additionally, we report the MR-Egger intercept and the MR pleiotropy residual sum and outlier (MR-PRESSO) global test as tests for horizontal pleiotropy. There were too few SNVs to use the secondary cataracts-GRS from GERA in 2-sample MR analyses. All analyses were conducted in R, version 1.4.1717 (R Project for Statistical Computing) and used the TwoSampleMR package, version 0.5.6. Data were analyzed from August 15, 2022, through November 28, 2023. Statistical tests were performed with an α = .05 and were 2-tailed and unpaired.

## Results

We included 308 272 participants aged 55 years or older in this analysis. We excluded individuals with dementia at baseline (n = 192 [0.06%]) or without self-reported visual history (n = 3127 [1.01%]). The full analytic sample included 304 953 participants (mean [SD] age, 62.06 [4.08] years; 163 825 women [53.72%]; 141 128 men [46.28%]). Smaller subsets of participants had available brain MRI data (ranging from 36 591 for total brain volume to 36 855 for lateral occipital volume) and/or had completed visual acuity examinations at initial assessment (69 852 for myopia and 71 429 for worse than 20/40 vision). During a mean (SD) follow-up of 12.71 (2.09) years, 7676 incident dementia cases were identified. When restricted to individuals with available data, 14 295 participants (4.69%) reported a history of cataracts, and 19 670 had myopia (28.16%), and 2754 (3.86%) had worse than 20/40 vision (Table 1). Those who reported cataracts were older at baseline (mean [SD], 63.94 [3.84] vs 61.96 [4.07] years) and more likely to be female (8505 [59.50%] vs 155 320 [53.44%]) compared with those without cataracts (n = 290 658).

**Table 1.** Characteristics of UK Biobank Participants Included in Analyses

Characteristic	No. with observed data	No. (%) or mean (SD)
Age, y	304 953	62.06 (4.08)
Sex	304 953	
Female	163 825 (53.72)	163 825 (53.72)
Male	141 128 (46.28)	141 128 (46.28)
Primary cataracts-GRS^a^	150 858	0.03 (0.98)
Myopia-GRS^a^	150 858	−0.02 (1)
AD-GRS with *APOE*^a^	183 439	0.01 (1)
Self-reported cataracts	304 953	14 295 (4.69)
Self-reported cataract surgery	304 953	2335 (0.77)
Self-reported glaucoma	304 953	6187 (2.03)
Self-reported AMD	304 953	3321 (1.09)
Self-reported diabetic retinopathy	304 953	2850 (0.93)
Myopia	69 g3	19 670 (28.16)
Worse than 20/40 vision	71 429	2754 (3.86)
Total brain volume, mm^3^	36 591	1 152 218.90 (109 824.54)
Lateral occipital volume, mm^3^	36 855	25 261.47 (3347.28)
Hippocampal volume, mm^3^	36 855	7998.23 (791.80)

Abbreviations: AD, Alzheimer disease; AMD, age-related macular degeneration; *APOE*, apolipoprotein E; GRS, genetic risk score.

^a^ See Methods for explanation of scoring.

### Associations With Dementia Risk

Cataracts were associated with all-cause dementia (hazard ratio [HR], 1.18; 95% CI, 1.07-1.29), AD (HR, 1.25; 95% CI, 1.07-1.47), and VaD (HR, 1.25; 95% CI, 1.01-1.55) (Table 2). When adjusted for cataract surgery (n = 2581 [0.5%]), cataracts were still associated with dementia (HR, 1.19; 95% CI, 1.08-1.32). Cataract surgery was not associated with dementia (odds ratio [OR], 0.99; 95% CI, 0.57-1.70), although 95% CIs were wide. There was no interaction between cataracts and cataract surgery (*P* = .72). Poor visual acuity was also associated with all-cause dementia (HR, 1.35; 95% CI, 1.06-1.70). However, myopia was not associated with dementia (HR, 0.92; 95% CI, 0.81-1.05) (Table 2). Glaucoma and age-related macular degeneration were not associated with dementia, although CIs were consistent with moderate increased risk. Diabetic retinopathy was associated with an increased hazard of all-cause dementia (HR, 1.63; 95% CI, 1.40-1.91) (eTable 6 in Supplement 1) and subtypes.

**Table 2.** Cox Proportional Hazards Regression Models for the Associations Between Eye Conditions and All-Cause Dementia, AD, and VaD^a^

Eye condition	No.	HR (95% CI)		
		All-cause dementia	AD	VaD
Cataracts	280 527	1.18 (1.07-1.29)	1.25 (1.07-1.47)	1.25 (1.01-1.55)
Myopia^b^	64 003	0.92 (0.81-1.05)	1.06 (0.85-1.34)	0.74 (0.50-1.11)
Binary 20/40 vision^b^	65 429	1.35 (1.06-1.70)	1.18 (0.75-1.85)	1.57 (0.85-2.90)

Abbreviations: AD, Alzheimer disease; HR, hazard ratio; VaD, vascular dementia.

^a^ Models include adjustment for age at visit, self-reported sex, self-reported racial and ethnic identity, index of multiple deprivation by country of origin, and binary indicators for history of falls, broken bones, cardiovascular disease, stroke, diabetes, and problems hearing.

^b^ Additionally adjusted for use of corrective lenses.

### Associations With Brain MRI Volumes

**Figure 2.**
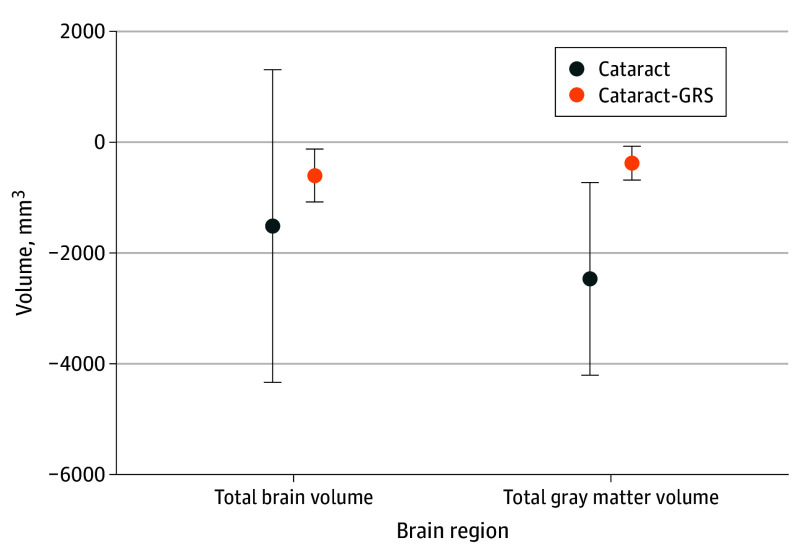
Association Between Cataracts or Cataracts-Genetic Risk Scores (GRS) and Total Brain and Gray Matter Volumes Models for cataracts include adjustment for age at visit, self-reported sex, self-reported racial and ethnic identity, index of multiple deprivation by country of origin, binary indicators for history of comorbidities (falls, broken bones, cardiovascular disease, stroke, diabetes, and problems hearing), and imaging center. Models for cataracts-GRS (primary) include adjustment for age, sex, first 10 principal components, imaging center, and intracranial volume. Error bars indicate 95% CI.

Cataracts were associated with smaller total gray matter volume (β = −2483.27 mm^3^; 95% CI, −4225.21 to −741.34 mm^3^) (Figure 2; eTable 7 in Supplement 1), smaller lateral occipital volume (β = −243.47 mm^3^; 95% CI, −416.81 to −70.12 mm^3^), and greater white matter hyperintensity volume (β = 531.00 mm^3^; 95% CI, 79.87-982.13 mm^3^). Cataracts-GRS was associated with smaller total gray matter volume (β = −375.17 mm^3^; 95% CI, −680.10 to −70.24 mm^3^) and total brain volume (β = −597.43 mm^3^; 95% CI, −1077.87 to −117.00 mm^3^). There was no evidence that genetic risk for cataracts was associated with AD-related cortical thinning (eTable 7 in Supplement 1).

### Mendelian Randomization

Genetic risk for cataracts (primary cataracts-GRS) was not associated with all-cause dementia (OR, 1.02; 95% CI, 0.99-1.05) or AD (OR, 1.00; 95% CI, 0.96-1.05), but it was associated with VaD (OR, 1.09; 95% CI, 1.03-1.16) (eTable 8 in Supplement 1). The secondary cataracts-GRS was associated with all-cause dementia (OR, 1.23; 95% CI, 1.20-1.26), AD (OR, 1.30; 95% CI, 1.25-1.35), and VaD (OR, 1.20; 95% CI, 1.14-1.26) (eTable 9 in Supplement 1). The myopia-GRS (primary and secondary) was not associated with any dementia outcome. In the reverse direction, AD-GRS was associated with lower odds of myopia (OR, 0.97; 95% CI, 0.95-0.99), but not with cataracts (OR, 0.99; 95% CI, 0.97-1.01) (eTable 10 in Supplement 1).

Using MR on our primary cataract-GRS, estimates for the possible causal association of cataracts with dementia were all greater than 1 (estimates ranging from 1.21 to 1.37) (Table 3), although no estimates were statistically significant. There was no evidence of horizontal pleiotropy, using the MR-Egger method (β = −0.01; *P* = .59). Similar estimates were noted with MR-PRESSO analysis (OR, 1.21; 95% CI, 0.98-1.50; global test for horizontal pleiotropy; *P* = .37).^45^ The association between individual cataract SNVs and all-cause dementia is shown in eFigure 1 in Supplement 1, with no evidence for large outliers. Mendelian randomization indicated greater odds of VaD with cataract (inverse variance weighted (OR, 1.92; 95% CI, 1.26-2.92, with all MR estimates ranging from 1.92 to 2.33) (Table 3), and all estimates were statistically significant except for MR-Egger (OR, 2.33; 95% CI, 0.90-6.02), which also provided no evidence of horizontal pleiotropy (intercept = −0.01; *P* = .66) (eFigure 2 in Supplement 1).

**Table 3.** MR Estimates for Vision Impairment and Dementia^a^

MR estimate	OR (95% CI)
**MR estimate of visual impairment association with dementia**	
Genetically predicted association between cataracts and all-cause dementia	
Inverse-variance weighted	1.21 (0.98-1.50)
MR-Egger	1.37 (0.84-2.23)
Weighted median	1.23 (0.91-1.66)
Weighted mode	1.21 (0.83-1.76)
Genetically predicted association between cataracts and vascular dementia	
IVW	1.92 (1.26-2.92)
MR-Egger	2.33 (0.90-6.02)
Weighted median	1.99 (1.05-3.77)
Weighted mode	2.24 (1.00-4.98)
**MR estimate of AD association with visual impairment (reverse causality)**	
Genetically predicted association between AD and self-reported and *ICD*-*10* diagnoses of cataracts	
IVW	0.99 (0.96-1.01)
MR-Egger	0.99 (0.96-1.03)
Weighted median	0.99 (0.97-1.01)
Weighted mode	1.00 (0.97-1.01)
Genetically predicted association between AD and myopia	
IVW	0.96 (0.92-1.00)
MR-Egger	0.98 (0.93-1.03)
Weighted median	0.96 (0.93-0.99)
Weighted mode	0.97 (0.94-1.01)
Genetically predicted association between AD and binary 20/40 vision	
IVW	0.93 (0.86-1.00)
MR-Egger	0.96 (0.88-1.05)
Weighted median	0.95 (0.88-1.03)
Weighted mode	0.95 (0.88-1.03)

Abbreviations: AD, Alzheimer disease; *ICD-10*, *International Statistical Classification of Diseases and Related Health Problems, 10th Revision*; IVW, inverse variance-weighted; MR, mendelian randomization; OR, odds ratio.

^a^ Models adjusted for age, sex, and first 10 principal components to proxy for genetic ancestry.

We checked for possible reverse causation and found that AD SNVs were not associated with cataracts (IVWOR, 0.99; 95% CI, 0.96-1.01), myopia, or binary 20/40 vision (eTable 11 in Supplement 1) across most summary MR estimates. There was no evidence of horizontal pleiotropy between AD and cataracts (β = −0.003; *P* = .39) (eFigure 3 in Supplement 1).

## Discussion

We evaluated vision impairments and dementia risk in UKB, using observational and MR approaches. Cataracts and poor visual acuity were associated with dementia risk. Higher genetic risk for cataracts was associated with increased risk of VaD. Analysis using 2-sample MR estimators showed cataracts increased VaD risk 2-fold. Genetic risk for AD was not associated with cataracts, suggesting observational associations are not due to reverse causation. We found evidence that cataracts may be associated with brain atrophy, specifically in global gray matter. In addition, myopia was associated with a decreased risk of dementia, but we found no evidence to support causality. Together, these results are consistent with the hypothesis that cataracts increase the risk of dementia.

Our results extend current evidence by observing that the association between cataracts and dementia may be causal and that cataracts are associated with brain volumes. Observational studies have found that cataracts were associated with increased dementia risk.^4,5,46,47^ Some previous work found no association between cataracts and dementia,^48,49^ but was underpowered. One MR study did not find a strong link between cataracts and AD^22^ but did not examine other dementia outcomes, such as VaD.

Our findings are strengthened by evaluating multiple ADRD-related outcomes, including neuroimaging markers. Few prior studies have examined associations between vision and ADRD-related neurodegeneration.^36,37,50^ In our study, cataracts were associated with increased brain atrophy, but we did not find evidence for AD-related neurodegeneration. Because a history of cataracts was associated with VaD and white matter hyperintensity volumes (a marker of small-vessel ischemic disease),^51^ there may be vascular mechanisms linking cataracts and dementia. Additionally, our observational work suggested an association between diabetic retinopathy and dementia. Future studies will need to confirm underlying mechanisms and should investigate other eye diseases (observationally and in MR studies), including diabetic retinopathy, optic neuropathy, and other vascular causes of eye disease.^52^ Future studies also should evaluate associations with ADRD pathologic burden, such as amyloid tau neurodegeneration biomarkers, alpha-synucleinopathies, and cerebrovascular markers.^53,54,55^

Our results are largely consistent with previous literature showing that poor visual acuity^6,7,8^ is associated with increased dementia risk. However, visual acuity was not associated with any neuroimaging measures, in contrast to another study.^36^ Myopia was associated with a decreased risk of dementia in observational models. Although this is consistent with some prior work,^56^ the finding is likely due to confounding, as the myopia-GRS was not associated with dementia.

The validity of our MR analyses depends on 4 assumptions.^20,21,43^ First, we confirmed that genetic instruments estimate each exposure of interest. Second, while we cannot prove that the independence assumption is met, we have adjusted for the strongest potential confounder: principal components.^57^ Third, we also believe the exclusion restriction assumption holds in our MR models, as there was no evidence for horizontal pleiotropy. Fourth, the monotonicity assumption limits the generalizability of our estimates and may not represent the true effect of cataracts on dementia in different populations.

### Strengths and Limitations

Our study has important strengths compared with prior work, including the strength of causal inference, a large sample, and an investigation into biological pathways.

This study has some important limitations. First, MR analyses are underpowered, which is reflected in the wide CIs of our MR estimates. However, by also including observational methods and multiple GRS, this study suggests there is a causal effect of cataracts on dementia by triangulation.^58^ Second, the genetic analyses were restricted to individuals with European ancestry, meaning genetic results may not be generalizable to other genetic backgrounds. Third, the present results do not consider monocular vs binocular presentation of cataracts, potential delays in cataract surgery by dementia status, or other factors. Fourth, the individuals in the UKB are relatively young (mean age, 62 years) and healthy,^59^ leading to only a smaller number of cases of ADRD. This study should be replicated in an older cohort with more diverse representation.

## Conclusions

In this cohort and bidirectional MR study of UKB participants, we added important insights into the link between eye conditions, visual impairment, and dementia risk by combining both observational and MR analyses. We found that cataracts, but not myopia, may increase the risk of dementia, especially VaD. Mendelian randomization and imaging findings suggested that non-AD biological pathways may explain this association. This study lends further support for the hypothesis that treatment or prevention of cataracts may prevent dementia. Clinical screening and treatment of cataracts may be important clinical strategies to improve quality of life. Future studies should test whether vision screening or cataract surgery in those with co-occurring dementia risk factors are effective at reducing the risk of dementia.

## Notes

Supplement 1. **eTable 1.** *International Classification of Diseases* (*ICD*) Codes Used to Extract Additional AD and ADRD diagnoses From Primary Care and In-Patient Electronic Health Records Through 2021 **eTable 2.** SNPs and Their Log OR Effect Estimates Included in the Primary Cataracts-GRS **eTable 3.** SNPs and Their Log OR Effect Estimates Included in the Primary Myopia-GRS **eTable 4.** SNPs and Their Log OR Effect Estimates Included in the AD-GRS **eTable 5.** Associations Between Genetic Risk Scores (Cataracts-GRS, Myopia-GRS, and AD-GRS) With Their Respective Outcomes in UK Biobank **eTable 6.** Cox Models for the Associations Between Glaucoma, AMD, and Diabetic Retinopathy With All-Cause Dementia, AD, and VaD **eTable 7.** Association Between Cataracts or Cataracts-GRS and Brain Regions (mm^3^) Associated With Dementia or Vision **eTable 8.** Association Between Primary GRS (Obtained From the 23andMe Replication Study) for Cataracts or Myopia, and Incident Dementia, AD, and VaD Among Participants Older Than 60 at Baseline **eTable 9.** Association Between Secondary GRS (Obtained From GERA Cohort) and Incident Dementia, AD, and VaD Among Participants Older Than 60 at Baseline **eTable 10.** Associations Between AD-GRS, With and Without *APOE*, and Vision Outcomes **eTable 11.** Summary Reverse Causation MR Estimates for: 1) the Association Between AD and Self-Reported and *ICD* Diagnoses of Cataracts, 2) the Association Between AD and Myopia, and 3) the Association Between AD and Binary 20/40 Vision **eFigure 1**. Association Between 43 Cataracts SNPs and Both Cataracts and Dementia **eFigure 2**. Association Between 43 Cataracts SNPs and Both Cataracts and Vascular Dementia **eFigure 3**. Association Between 27 AD SNPs and Both Cataracts and Dementia

Supplement 2.
Data Sharing Statement

## References

[1] Ehrlich JR, Goldstein J, Swenor BK, Whitson H, Langa KM, Veliz P. Addition of vision impairment to a life-course model of potentially modifiable dementia risk factors in the US. JAMA Neurol. 2022;79(6):623-626. doi:10.1001/jamaneurol.2022.0723 35467745 PMC9039828

[2] Shang X, Zhu Z, Wang W, Ha J, He M. The association between vision impairment and incidence of dementia and cognitive impairment: a systematic review and meta-analysis. Ophthalmology. 2021;128(8):1135-1149. doi:10.1016/j.ophtha.2020.12.029 33422559

[3] Hwang PH, Longstreth WT Jr, Thielke SM, . longitudinal changes in hearing and visual impairments and risk of dementia in older adults in the United States. JAMA Netw Open. 2022;5(5):e2210734. doi:10.1001/jamanetworkopen.2022.10734 35511175 PMC9073563

[4] Ma LZ, Zhang YR, Li YZ, . Cataract, cataract surgery, and risk of incident dementia: a prospective cohort study of 300,823 participants. Biol Psychiatry. 2023;93(9):810-819. doi:10.1016/j.biopsych.2022.06.005 35940935

[5] Hwang PH, Longstreth WT Jr, Thielke SM, . Ophthalmic conditions associated with dementia risk: the Cardiovascular Health Study. Alzheimers Dement. 2021;17(9):1442-1451. doi:10.1002/alz.12313 33788406 PMC8527838

[6] Lee ATC, Richards M, Chan WC, Chiu HFK, Lee RSY, Lam LCW. Higher dementia incidence in older adults with poor visual acuity. J Gerontol A Biol Sci Med Sci. 2020;75(11):2162-2168. doi:10.1093/gerona/glaa036 32043518 PMC7566398

[7] Elyashiv SM, Shabtai EL, Belkin M. Correlation between visual acuity and cognitive functions. Br J Ophthalmol. 2014;98(1):129-132. doi:10.1136/bjophthalmol-2013-304149 24169658 PMC4060517

[8] Zhu Z, Shi D, Liao H, . Visual impairment and risk of dementia: the UK Biobank Study. Am J Ophthalmol. 2022;235:7-14. doi:10.1016/j.ajo.2021.08.010 34433084

[9] Lee CS, Gibbons LE, Lee AY, . Association between cataract extraction and development of dementia. JAMA Intern Med. 2022;182(2):134-141. doi:10.1001/jamainternmed.2021.6990 34870676 PMC8649913

[10] Swenor BK, Lee MJ, Varadaraj V, Whitson HE, Ramulu PY. Aging with vision loss: a framework for assessing the impact of visual impairment on older adults. Gerontologist. 2020;60(6):989-995. doi:10.1093/geront/gnz117 31504483 PMC7427480

[11] Mendez I, Kim M, Lundeen EA, Loustalot F, Fang J, Saaddine J. Cardiovascular disease risk factors in US adults with vision impairment. Prev Chronic Dis. 2022;19:E43. doi:10.5888/pcd19.220027 35862513 PMC9336192

[12] Vu TA, Fenwick EK, Gan ATL, . The bidirectional relationship between vision and cognition: a systematic review and meta-analysis. Ophthalmology. 2021;128(7):981-992. doi:10.1016/j.ophtha.2020.12.010 33333104 PMC12962451

[13] Cook LA, Sachs J, Weiskopf NG. The quality of social determinants data in the electronic health record: a systematic review. J Am Med Inform Assoc. 2021;29(1):187-196. doi:10.1093/jamia/ocab199 34664641 PMC8714289

[14] Adler NE, Stead WW. Patients in context—EHR capture of social and behavioral determinants of health. N Engl J Med. 2015;372(8):698-701. doi:10.1056/NEJMp1413945 25693009

[15] Majoka MA, Schimming C. Effect of social determinants of health on cognition and risk of Alzheimer disease and related dementias. Clin Ther. 2021;43(6):922-929. doi:10.1016/j.clinthera.2021.05.005 34103175

[16] Kyriacou DN, Lewis RJ. Confounding by indication in clinical research. JAMA. 2016;316(17):1818-1819. doi:10.1001/jama.2016.16435 27802529

[17] Kunkle BW, Grenier-Boley B, Sims R, ; Alzheimer Disease Genetics Consortium (ADGC); European Alzheimer’s Disease Initiative (EADI); Cohorts for Heart and Aging Research in Genomic Epidemiology Consortium (CHARGE); Genetic and Environmental Risk in AD/Defining Genetic, Polygenic and Environmental Risk for Alzheimer’s Disease Consortium (GERAD/PERADES). Genetic meta-analysis of diagnosed Alzheimer’s disease identifies new risk loci and implicates Aβ, tau, immunity and lipid processing. Nat Genet. 2019;51(3):414-430. doi:10.1038/s41588-019-0358-2 30820047 PMC6463297

[18] Choquet H, Melles RB, Anand D, ; 23andMe Research Team. A large multiethnic GWAS meta-analysis of cataract identifies new risk loci and sex-specific effects. Nat Commun. 2021;12(1):3595. doi:10.1038/s41467-021-23873-8 34127677 PMC8203611

[19] Hysi PG, Choquet H, Khawaja AP, ; Consortium for Refractive Error and Myopia; UK Eye and Vision Consortium; 23andMe Inc. Meta-analysis of 542,934 subjects of European ancestry identifies new genes and mechanisms predisposing to refractive error and myopia. Nat Genet. 2020;52(4):401-407. doi:10.1038/s41588-020-0599-0 32231278 PMC7145443

[20] Emdin CA, Khera AV, Kathiresan S. Mendelian randomization. JAMA. 2017;318(19):1925-1926. doi:10.1001/jama.2017.17219 29164242

[21] Glymour MM, Tchetgen Tchetgen EJ, Robins JM. Credible mendelian randomization studies: approaches for evaluating the instrumental variable assumptions. Am J Epidemiol. 2012;175(4):332-339. doi:10.1093/aje/kwr323 22247045 PMC3366596

[22] Man S, Chen B, Zhang Y, . The associations between cataracts and Alzheimer’s disease: a bidirectional two-sample mendelian randomization study. J Alzheimers Dis. 2023;92(4):1451-1458. doi:10.3233/JAD-221137 36911941

[23] Deal J, Rojas JC. Visual impairment as a modifiable risk factor in dementia prevention and management. JAMA Neurol. 2022;79(6):542-543. doi:10.1001/jamaneurol.2022.0092 35467705 PMC9949617

[24] Sudlow C, Gallacher J, Allen N, . UK Biobank: an open access resource for identifying the causes of a wide range of complex diseases of middle and old age. PLoS Med. 2015;12(3):e1001779. doi:10.1371/journal.pmed.1001779 25826379 PMC4380465

[25] Skrivankova VW, Richmond RC, Woolf BAR, . Strengthening the Reporting of Observational Studies in Epidemiology Using Mendelian Randomization: the STROBE-MR statement. JAMA. 2021;326(16):1614-1621. doi:10.1001/jama.2021.18236 34698778

[26] Guggenheim JA, Williams C; UK Biobank Eye and Vision Consortium. Role of educational exposure in the association between myopia and birth order. JAMA Ophthalmol. 2015;133(12):1408-1414. doi:10.1001/jamaophthalmol.2015.3556 26448589 PMC4681114

[27] Chua SYL, Thomas D, Allen N, ; UK Biobank Eye & Vision Consortium. Cohort profile: design and methods in the eye and vision consortium of UK Biobank. BMJ Open. 2019;9(2):e025077. doi:10.1136/bmjopen-2018-025077 30796124 PMC6398663

[28] Wilkinson T, Schnier C, Bush K, ; Dementias Platform UK and UK Biobank. Identifying dementia outcomes in UK Biobank: a validation study of primary care, hospital admissions and mortality data. Eur J Epidemiol. 2019;34(6):557-565. doi:10.1007/s10654-019-00499-1 30806901 PMC6497624

[29] UK Biobank. Algorithmically-defined outcomes (ADOs). January 2022. Accessed April 19, 2023. https://biobank.ndph.ox.ac.uk/showcase/ukb/docs/alg_outcome_main.pdf

[30] Aljondi R, Szoeke C, Steward C, Yates P, Desmond P. A decade of changes in brain volume and cognition. Brain Imaging Behav. 2019;13(2):554-563. doi:10.1007/s11682-018-9887-z 29744801

[31] Jagust WJ, Zheng L, Harvey DJ, . Neuropathological basis of magnetic resonance images in aging and dementia. Ann Neurol. 2008;63(1):72-80. doi:10.1002/ana.21296 18157909 PMC2624571

[32] Honea RA, Swerdlow RH, Vidoni ED, Goodwin J, Burns JM. Reduced gray matter volume in normal adults with a maternal family history of Alzheimer disease. Neurology. 2010;74(2):113-120. doi:10.1212/WNL.0b013e3181c918cb 20065246 PMC2809030

[33] Wolf H, Grunwald M, Kruggel F, . Hippocampal volume discriminates between normal cognition; questionable and mild dementia in the elderly. Neurobiol Aging. 2001;22(2):177-186. doi:10.1016/S0197-4580(00)00238-4 11182467

[34] Smith EE, Egorova S, Blacker D, . Magnetic resonance imaging white matter hyperintensities and brain volume in the prediction of mild cognitive impairment and dementia. Arch Neurol. 2008;65(1):94-100. doi:10.1001/archneurol.2007.23 18195145

[35] Schwarz CG, Gunter JL, Wiste HJ, ; Alzheimer’s Disease Neuroimaging Initiative. A large-scale comparison of cortical thickness and volume methods for measuring Alzheimer’s disease severity. Neuroimage Clin. 2016;11:802-812. doi:10.1016/j.nicl.2016.05.017 28050342 PMC5187496

[36] Garzone D, Finger RP, Mauschitz MM, . Visual impairment and retinal and brain neurodegeneration: a population-based study. Hum Brain Mapp. 2023;44(7):2701-2711. doi:10.1002/hbm.26237 36852616 PMC10089094

[37] Marchesi N, Fahmideh F, Boschi F, Pascale A, Barbieri A. Ocular neurodegenerative diseases: interconnection between retina and cortical areas. Cells. 2021;10(9):2394. doi:10.3390/cells10092394 34572041 PMC8469605

[38] Littlejohns TJ, Holliday J, Gibson LM, . The UK Biobank imaging enhancement of 100,000 participants: rationale, data collection, management and future directions. Nat Commun. 2020;11(1):2624. doi:10.1038/s41467-020-15948-9 32457287 PMC7250878

[39] Bycroft C, Freeman C, Petkova D, . The UK Biobank resource with deep phenotyping and genomic data. Nature. 2018;562(7726):203-209. doi:10.1038/s41586-018-0579-z 30305743 PMC6786975

[40] UK Biobank. Genotyping and quality control of UK Biobank, a large-scale, extensively phenotyped prospective resource. 2015. Accessed April 24, 2023. https://biobank.ctsu.ox.ac.uk/crystal/crystal/docs/genotyping_qc.pdf

[41] Choi SW, Mak TSH, O’Reilly PF. Tutorial: a guide to performing polygenic risk score analyses. Nat Protoc. 2020;15(9):2759-2772. doi:10.1038/s41596-020-0353-1 32709988 PMC7612115

[42] Burgess S, Davies NM, Thompson SG. Bias due to participant overlap in two-sample mendelian randomization. Genet Epidemiol. 2016;40(7):597-608. doi:10.1002/gepi.21998 27625185 PMC5082560

[43] Burgess S, Davey Smith G, Davies NM, . Guidelines for performing mendelian randomization investigations: update for summer 2023. Wellcome Open Res. 2023;4:186. doi:10.12688/wellcomeopenres.15555.3 32760811 PMC7384151

[44] Burgess S, Labrecque JA. Mendelian randomization with a binary exposure variable: interpretation and presentation of causal estimates. Eur J Epidemiol. 2018;33(10):947-952. doi:10.1007/s10654-018-0424-6 30039250 PMC6153517

[45] Verbanck M, Chen CY, Neale B, Do R. Detection of widespread horizontal pleiotropy in causal relationships inferred from mendelian randomization between complex traits and diseases. Nat Genet. 2018;50(5):693-698. doi:10.1038/s41588-018-0099-7 29686387 PMC6083837

[46] Shang X, Zhu Z, Huang Y, . Associations of ophthalmic and systemic conditions with incident dementia in the UK Biobank. Br J Ophthalmol. 2023;107(2):275-282. doi:10.1136/bjophthalmol-2021-319508 34518160

[47] Xiong Z, Li X, Yang D, Xiong C, Xu Q, Zhou Q. The association between cataract and incidence of cognitive impairment in older adults: a systematic review and meta-analysis. Behav Brain Res. 2023;450:114455. doi:10.1016/j.bbr.2023.114455 37148915

[48] Xiao Z, Wu W, Zhao Q, Liang X, Luo J, Ding D. Association of glaucoma and cataract with incident dementia: a 5-year follow-up in the Shanghai Aging Study. J Alzheimers Dis. 2020;76(2):529-537. doi:10.3233/JAD-200295 32538850

[49] Lee CS, Larson EB, Gibbons LE, . Associations between recent and established ophthalmic conditions and risk of Alzheimer’s disease. Alzheimers Dement. 2019;15(1):34-41. doi:10.1016/j.jalz.2018.06.2856 30098888 PMC6333518

[50] Prins D, Hanekamp S, Cornelissen FW. Structural brain MRI studies in eye diseases: are they clinically relevant? a review of current findings. Acta Ophthalmol. 2016;94(2):113-121. doi:10.1111/aos.12825 26361248

[51] Pantoni L, Poggesi A, Inzitari D. The relation between white-matter lesions and cognition. Curr Opin Neurol. 2007;20(4):390-397. doi:10.1097/WCO.0b013e328172d661 17620872

[52] Chatziralli IP, Kazantzis D, Chatzirallis AP, . Cardiometabolic factors and risk of non-arteritic anterior ischemic optic neuropathy: a systematic review and meta-analysis. Graefes Arch Clin Exp Ophthalmol. 2022;260(5):1445-1456. doi:10.1007/s00417-021-05522-4 35067769

[53] Wang L, Benzinger TL, Su Y, . Evaluation of tau imaging in staging Alzheimer disease and revealing interactions between β-amyloid and tauopathy. JAMA Neurol. 2016;73(9):1070-1077. doi:10.1001/jamaneurol.2016.2078 27454922 PMC5237382

[54] Moore EE, Gifford KA, Khan OA, . Cerebrospinal fluid biomarkers of neurodegeneration, synaptic dysfunction, and axonal injury relate to atrophy in structural brain regions specific to Alzheimer’s disease. Alzheimers Dement. 2020;16(6):883-895. doi:10.1002/alz.12087 32378327 PMC7781154

[55] Moore EE, Khan OA, Liu D, . Baseline cerebrospinal fluid biomarkers of amyloidosis, phosphorylated tau, and total tau relate to greater longitudinal atrophy in regions susceptible to Alzheimer’s disease–related neurodegeneration. Alzheimers Dement. 2020;16(S5):e046095. doi:10.1002/alz.046095

[56] Spierer O, Fischer N, Barak A, Belkin M. Correlation between vision and cognitive function in the elderly: a cross-sectional study. Medicine (Baltimore). 2016;95(3):e2423. doi:10.1097/MD.0000000000002423 26817872 PMC4998246

[57] Davies NM, Holmes MV, Davey Smith G. Reading Mendelian randomisation studies: a guide, glossary, and checklist for clinicians. BMJ. 2018;362:k601. doi:10.1136/bmj.k601 30002074 PMC6041728

[58] Lawlor DA, Tilling K, Davey Smith G. Triangulation in aetiological epidemiology. Int J Epidemiol. 2016;45(6):1866-1886. 28108528 10.1093/ije/dyw314 PMC5841843

[59] Fry A, Littlejohns TJ, Sudlow C, . Comparison of sociodemographic and health-related characteristics of UK Biobank participants with those of the general population. Am J Epidemiol. 2017;186(9):1026-1034. doi:10.1093/aje/kwx246 28641372 PMC5860371

[60] Besagar S, Yonekawa Y, Sridhar J, et al. Association of socioeconomic, demographic, and health care access disparities with severe visual impairment in the US. JAMA Ophthalmol. 2022;140(12):1219-1226. doi:10.1001/jamaophthalmol.2022.4566 PMC9634598 36326732

[61] Balls-Berry JJE, Babulal GM. Health disparities in dementia. Continuum (Minneap Minn). 2022;28(3):872-884. doi:10.1212/CON.0000000000001088 PMC9924306 35678407

