# Supplementary material for: Visual Impairment, Eye Conditions, and Diagnoses of Neurodegeneration and Dementia
Source: JAMA Netw Open. 2024 Jul 30;7(7):e2424539. doi: 10.1001/jamanetworkopen.2024.24539 (PMC11289698; doi:10.1001/jamanetworkopen.2024.24539)
Supplement: Supplement 1. — eTable 1. International Classification of Diseases (ICD) Codes Used to Extract Additional AD and ADRD diagnoses From Primary Care and In-Patient Electronic Health Records Through 2021 eTable 2. SNPs and Their Log OR Effect Estimates Included in the Primary Cataracts-GRS eTable 3. SNPs and Their Log OR Effect Estimates Included in the Primary Myopia-GRS eTable 4. SNPs and Their Log OR Effect Estimates Included in the AD-GRS eTable 5. Associations Between Genetic Risk Scores (Cataracts-GRS, Myopia-GRS, and AD-GRS) With Their Respective Outcomes in UK Biobank eTable 6. Cox Models for the Associations Between Glaucoma, AMD, and Diabetic Retinopathy With All-Cause Dementia, AD, and VaD eTable 7. Association Between Cataracts or Cataracts-GRS and Brain Regions (mm3) Associated With Dementia or Vision eTable 8. Association Between Primary GRS (Obtained From the 23andMe Replication Study) for Cataracts or Myopia, and Incident Dementia, AD, and VaD Among Participants Older Than 60 at Baseline eTable 9. Association Between Secondary GRS (Obtained From GERA Cohort) and Incident Dementia, AD, and VaD Among Participants Older Than 60 at Baseline eTable 10. Associations Between AD-GRS, With and Without APOE, and Vision Outcomes eTable 11. Summary Reverse Causation MR Estimates for: 1) the Association Between AD and Self-Reported and ICD Diagnoses of Cataracts, 2) the Association Between AD and Myopia, and 3) the Association Between AD and Binary 20/40 Vision eFigure 1. Association Between 43 Cataracts SNPs and Both Cataracts and Dementia eFigure 2. Association Between 43 Cataracts SNPs and Both Cataracts and Vascular Dementia eFigure 3. Association Between 27 AD SNPs and Both Cataracts and Dementia [file jamanetwopen-e2424539-s001.pdf]

Supplementary Online Content

Ferguson EL, Thoma M, Buto PT, et al. Visual impairment, eye conditions, and diagnoses of neurodegeneration and dementia. *JAMA Netw Open*. 2024;7(7):e2424539. doi:10.1001/jamanetworkopen.2024.24539

eTable 1. *International Classification of Diseases* (*ICD*) Codes Used to Extract Additional AD and ADRD diagnoses From Primary Care and In-Patient Electronic Health Records Through 2021

eTable 2. SNPs and Their Log OR Effect Estimates Included in the Primary Cataracts-GRS

eTable 3. SNPs and Their Log OR Effect Estimates Included in the Primary Myopia-GRS.

eTable 4. SNPs and Their Log OR Effect Estimates Included in the AD-GRS

eTable 5. Associations Between Genetic Risk Scores (Cataracts-GRS, Myopia-GRS, and AD-GRS) With Their Respective Outcomes in UK Biobank

eTable 6. Cox Models for the Associations Between Glaucoma, AMD, and Diabetic Retinopathy With All-Cause Dementia, AD, and VaD

eTable 7. Association Between Cataracts or Cataracts-GRS and Brain Regions (mm^3) Associated With Dementia or Vision

eTable 8. Association Between Primary GRS (Obtained From the 23andMe Replication Study) for Cataracts or Myopia, and Incident Dementia, AD, and VaD Among Participants Older Than 60 at Baseline

eTable 9. Association Between Secondary GRS (Obtained From GERA Cohort) and Incident Dementia, AD, and VaD Among Participants Older Than 60 at Baseline

eTable 10. Associations Between AD-GRS, With and Without *APOE*, and Vision Outcomes

eTable 11. Summary Reverse Causation MR Estimates for: 1) the Association Between AD and Self-Reported and *ICD* Diagnoses of Cataracts, 2) the Association Between AD and Myopia, and 3) the Association Between AD and Binary 20/40 Vision

eFigure 1. Association Between 43 Cataracts SNPs and Both Cataracts and Dementia

eFigure 2. Association Between 43 Cataracts SNPs and Both Cataracts and Vascular Dementia

eFigure 3. Association Between 27 AD SNPs and Both Cataracts and Dementia

This supplementary material has been provided by the authors to give readers additional information about their work.

© 2024 Ferguson EL et al. *JAMA Network Open.*

eTable 1. ***International Classification of Diseases (ICD)* codes used to extract additional AD and ADRD diagnoses from primary care and in-patient electronic health records through 2021.**

**Description**	***ICD-10* code**	**Subtype**
Dementia in Alzheimer's disease	F00	AD
Dementia in Alzheimer's disease with early onset	F00.0	AD
Dementia in Alzheimer's disease with late onset	F00.1	AD
Dementia in Alzheimer's disease, atypical or mixed type	F00.2	AD
Dementia in Alzheimer's disease, unspecified	F00.9	AD
Alzheimer’s disease	G30	AD
Alzheimer’s disease with early onset	G30.0	AD
Alzheimer’s disease with late onset	G30.1	AD
Other Alzheimer's disease	G30.8	AD
Alzheimer's disease unspecified	G30.9	AD
Vascular dementia	F01	ADRD
Vascular dementia of acute onset	F01.0	ADRD
Multi-infarct dementia	F01.1	ADRD
Subcortical vascular dementia	F01.2	ADRD
Mixed cortical and sub-cortical vascular dementia	F01.3	ADRD
Other vascular dementia	F01.8	ADRD
Vascular dementia, unspecified	F01.9	ADRD
Binswanger's disease	I67.3	ADRD
Dementia in Picks disease	F02.0	ADRD
Circumscribed brain atrophy	G31.0	ADRD
Sporadic Creutzfeldt-Jakob disease	A81.0	ADRD
Dementia in Creutzfeldt-Jacob disease	F02.1	ADRD
Dementia in Huntington’s disease	F02.2	ADRD
Dementia in Parkinson’s disease	F02.3	ADRD
Dementia in HIV disease	F02.4	ADRD
Mental and behavioural disorders due to use of alcohol - amnesic syndrome	F10.6	ADRD
Dementia in other diseases classified elsewhere	F02	ADRD
Dementia in other specified diseases classified elsewhere	F02.8	ADRD
Unspecified dementia	F03	ADRD
Delirium superimposed on dementia	F05.1	ADRD
Senile degeneration of brain	G31.1	ADRD
Other specified degenerative diseases of nervous system	G31.8	ADRD

Abbreviations: ADRD = Alzheimer disease and related dementias; AD = Alzheimer disease

© 2024 Ferguson EL et al. *JAMA Network Open.*

eTable 2. SNPs and their log OR effect estimates included in the primary Cataracts-GRS.

Variant	Chromosome	Locus	Effect Allele	Effect Estimate
rs2073017	1	*CASZ1*	C	-0.0202027
rs3176459	1	*CDKN2C*	G	-0.0202027
rs71646944	1	*ADGRL2*	T	0.03922071
rs10633030	1	*FAM46C*	CT	0.0295588
rs2982459	1	*LINC00970*	G	-0.0304592
rs12593	1	*ADCK3*	T	0.03922071
rs890069	2	*Near TRIB2*	T	-0.0202027
rs10210444	2	*PLB1*	A	0.03922071
rs7604689	2	*LRP1B-KYNU*	C	-0.0304592
rs62237590	3	*RARB*	C	0.01980263
rs35256080	3	*ATXN7*	AT	0.01980263
rs10663094	3	*SOX2-OT*	ACT	0.04879016
rs72868578	4	*C4orf22-BMP3*	A	0.06765865
rs7744813	6	*KCNQ5*	A	0.01980263
rs73015318	6	*QKI*	A	0.04879016
rs10258092	7	*CREB5*	C	0.0295588
rs17172647	7	*IGFBP3-TNS3*	G	0.06765865
rs62621812	7	*ZNF800*	A	0.11332869
rs12114462	8	*BIN3-EGR3*	C	0.01980263
rs1679013	9	*CDKN2B-DMRTA1*	T	0.0295588
rs4742654	9	*FKTN-TAL2*	T	0.01980263
rs4837205	9	*ST6GALNAC4-PIP5KL1*	C	-0.0202027
rs1014607	10	*BAMBI-LINC01517*	A	-0.040822
rs2274224	10	*PLCE1*	C	0.0295588
rs73386631	11	*ODF3-BET1L*	T	0.05826891
rs150648223	11	*5' LOC338694*	ATTT	0.09531018
rs17739338	12	*CAPRIN2*	T	-0.0618754
rs17608087	12	*MVK-FAM222A*	G	0.04879016
rs7154613	14	*STXBP6*	T	0.0295588
rs2855530	14	*BMP4*	C	0.0295588
rs72714121	15	*OCA2*	T	0.04879016
rs12901945	15	*RORA-VPS13C*	A	0.01980263
rs10500355	16	*RBFOX1*	A	0.03922071
rs73530148	16	*ALDOA*	T	0.03922071
rs73568154	16	*WWP2*	A	0.01980263
rs8074331	17	*RHOT1 - RHBDL3*	A	-0.0202027

© 2024 Ferguson EL et al. *JAMA Network Open.*

Variant	Chromosome	Locus	Effect Allele	Effect Estimate
rs7207025	17	*near MIR2117HG*	A	-0.0202027
rs9038	17	*9-Sep*	C	-0.040822
rs9895741	17	*NPLOC4*	G	-0.0512933
rs75954926	17	*3′ METRNL*	G	0.0295588
rs61744414	19	*CPAMD8*	T	0.09531018
rs549768142	20	*JAG1*	GAAAAAAAAAAT	-0.040822
rs4814857	20	*SLC24A3*	G	0.0861777

Abbreviations: GRS = genetic risk score; SNP = single nucleotide polymorphism

© 2024 Ferguson EL et al. *JAMA Network Open.*

eTable 3. SNPs and their log OR effect estimates included in the primary Myopia-GRS.

Variant	Chromosome	Position	Effect Allele	Effect Estimate
rs2808515	1	200346324	G	0.0707
rs2017760	1	207455421	G	-0.1036
rs300751	2	209063	C	-0.0774
rs9309272	2	56095366	T	0.127
rs61049169	2	146888708	A	-0.0755
rs192716	2	157376472	C	-0.0829
rs2573081	2	178828507	G	0.0646
rs1550094	2	233385396	A	-0.1004
rs14165	3	53847408	G	0.0779
rs6549043	3	85632126	T	0.0739
rs6767786	3	141104180	A	-0.0664
rs74764079	4	81952637	A	0.2958
rs1309551	5	64288656	G	-0.0693
rs7730838	5	71708698	T	-0.0627
rs7444298	5	87730027	G	0.0846
rs11758482	6	2438231	G	0.0927
rs4145443	6	22068174	T	0.0729
rs2207136	6	50809720	C	-0.0689
rs7744813	6	73643289	A	0.1006
rs12193446	6	129820038	G	-0.2244
rs6465760	7	99591418	A	0.069
rs12234576	7	158928375	A	-0.1062
rs16890057	8	40726582	A	-0.0794
rs72621438	8	60178580	G	-0.1064
rs9643433	8	78952371	A	-0.0708
rs62538956	9	12679244	C	0.1016
rs11145746	9	71834380	A	0.0969
rs7042950	9	77149837	G	0.0919
rs10826198	10	60274182	T	0.0731
rs12778014	10	94950273	A	-0.0835
rs807037	10	102824349	C	0.0695
rs56299331	10	114788436	T	0.0811
rs6484385	11	28668416	C	0.0744
rs11602008	11	40149305	T	0.1204
rs11226856	11	105699048	G	0.0918
rs5442	12	6954864	A	0.1439

© 2024 Ferguson EL et al. *JAMA Network Open.*

Variant	Chromosome	Position	Effect Allele	Effect Estimate
rs1468993	12	46155145	C	0.0786
rs3138142	12	56115585	T	-0.1191
rs11178462	12	71269789	A	-0.1055
rs1328371	13	93912785	C	-0.066
rs34217772	14	42273570	G	0.0967
rs34935520	14	61091401	A	-0.0772
rs35337422	14	104407243	C	0.1109
rs524952	15	35005886	A	0.1713
rs75227249	15	48763008	T	-0.1161
rs1007365	15	79379492	G	0.0853
rs8039459	15	82320426	G	0.0755
rs17648524	16	7459683	C	0.0878
rs4635359	16	80537760	C	-0.078
rs2908972	17	11407259	A	0.0886
rs62067167	17	31251711	T	0.1156
rs7222840	17	47280915	C	0.0851
rs12603264	17	54729518	T	0.0765
rs4793501	17	68718734	T	0.0996
rs734559	18	72179579	A	-0.0811

Abbreviations: GRS = genetic risk score; SNP = single nucleotide polymorphism

© 2024 Ferguson EL et al. *JAMA Network Open.*

eTable 4. SNPs and their log OR effect estimates included in the AD-GRS.

Variant	Chromosome	Locus	Effect Allele	Effect Estimate
rs4844610	1	*CR1*	A	0.157
rs6733839	2	*BIN1*	T	0.182
rs10933431	2	*INPP5D*	G	-0.094
rs9271058	6	*HLA -DRB1*	A	0.095
rs75932628	6	*TREM2*	T	0.732
rs9473117	6	*CD2AP*	C	0.086
rs114812713	6	*OARD1*	C	0.278
rs12539172	7	*NYAP1*	T	-0.083
rs10808026	7	*EPHA1*	A	-0.105
rs73223431	8	*PTK2B*	T	0.095
rs9331896	8	*CLU*	C	-0.128
rs7920721	10	*ECHDC3*	G	0.077
rs3740688	11	*SPI1*	G	-0.083
rs7933202	11	*MS4A2*	C	-0.117
rs3851179	11	*PICALM*	T	-0.128
rs11218343	11	*SORL1*	C	-0.223
rs17125924	14	*FERMT2*	G	0.131
rs12881735	14	*SLC24A4*	C	-0.083
rs593742	15	*ADAM10*	G	-0.073
rs7185636	16	*IQCK*	C	-0.083
rs62039712	16	*WWOX*	A	0.148
rs138190086	17	*ACE*	A	0.262
rs3752246	19	*ABCA7*	G	0.140
rs429358	19	*APOE*	C	1.351
rs7412	19	*APOE*	T	-0.386
rs6024870	20	*CASS4*	A	-0.128
rs2830500	21	*ADAMTS1*	A	-0.073

Abbreviations: GRS = genetic risk score; SNP = single nucleotide polymorphism

© 2024 Ferguson EL et al. *JAMA Network Open.*

eTable 5. Associations between genetic risk scores (Cataracts-GRS, Myopia-GRS, and AD-GRS) with their respective outcomes in UK Biobank. Models include adjustment for age, sex, and first 10 principal components.

Genetic Risk Score	Outcome	n	Adjusted OR (95% CI)
Primary Analysis			
Primary Cataracts-GRS	Self-Reported Cataracts	150,858	1.18 (1.16-1.21)
Primary Cataracts-GRS	Self-Reported and ICD Diagnoses of Cataracts	150,858	1.21 (1.19-1.23)
Primary Myopia-GRS	Assessment-Derived Myopia	34,157	1.44 (1.40-1.47
AD-GRS	Incident ADRD	183,439	1.80 (1.76-1.84)
Secondary Analyses			
Secondary Cataracts-GRS	Self-Reported Cataracts	183,439	1.05 (1.03-1.07)
Secondary Cataracts-GRS	Self-Reported and ICD Diagnoses of Cataracts	183,439	1.05 (1.04-1.06)
Secondary Myopia-GRS	Assessment-Derived Myopia	40,665	0.72 (0.71-0.74)
AD-GRS without *APOE*	Incident ADRD	183,439	1.20 (1.17-1.24)

Abbreviations: GRS = genetic risk score; ADRD = Alzheimer disease and related dementias; AD = Alzheimer disease, *APOE* = apolipoprotein E (*APOE*) gene

© 2024 Ferguson EL et al. *JAMA Network Open.*

eTable 6. Cox models for the associations between glaucoma, AMD, and diabetic retinopathy with all-cause dementia, AD, and VaD. Models include adjustment for: age at visit, self-reported sex, self-reported racial and ethnic identity, index of multiple deprivation by country of origin, and binary indicators for history of falls, broken bones, cardiovascular disease, stroke, diabetes, and problems hearing.

Eye condition	n	All-Cause Dementia HR (95% CI)	AD HR (95% CI)	VaD HR (95% CI)
Glaucoma	280,527	1.13 (0.98-1.30)	1.00 (0.76-1.30)	1.25 (0.91-1.74)
Age-related Macular Degeneration	280,527	1.17 (0.97-1.41)	0.94 (0.65-1.35)	1.25 (0.80-1.95)
Diabetic Retinopathy	280,527	1.63 (1.40-1.91)	1.41 (1.00-1.97)	1.77 (1.30-2.41)

Abbreviations: AD = Alzheimer disease, AMD = age-related macular degeneration, VaD = vascular dementia

© 2024 Ferguson EL et al. *JAMA Network Open.*

eTable 7. Association between cataracts or cataracts-GRS and brain regions (mm³) associated with dementia or vision. Models for cataracts include adjustment for: age at visit, self-reported sex, self-reported racial and ethnic identity, index of multiple deprivation by country of origin, binary indicators for history of comorbidities (falls, broken bones, cardiovascular disease, stroke, diabetes, and problems hearing), and imaging center. Models for cataracts-GRS include adjustment for: age, sex, first 10 principal components, and imaging center.

**Exposure**	**Brain Region**	**Coefficient (mm³) (95% CI)**
Self-reported Cataracts (n=33,271)	Total brain volume[a]	-1515.33 (-4351.56, 1320.91)
	Total grey matter volume[a]	**-2483.27 (-4225.21, -741.34)**
	Hippocampal volume[a]	-27.11 (-69.47, 15.25)
	White matter hyperintensity volume[a]	**531.00 (79.87, 982.13)**
	AD-Signature Region	-0.01 (-0.01, 7e-04)
	Lateral occipital volume[a]	**-243.47 (-416.81, -70.12)**
Cataracts-GRS (n= 26,051)	Total brain volume[a]	**-597.432 (-1077.87, -117.00)**
	Total grey matter volume[a]	**-375.17 (-680.10, -70.24)**
	Hippocampal volume[a]	-4.28 (-11.90, 3.33)
	White matter hyperintensity volume[a]	7.60 (-75.67, 90.88)
	AD-Signature Region	-0.0005 (-0.002, 8e-04)
	Lateral occipital volume[a]	26.45 (-6.21, 49.75)

[a]Additionally adjusted for intracranial volume.

Abbreviations: GRS = genetic risk score; AD = Alzheimer disease

© 2024 Ferguson EL et al. *JAMA Network Open.*

eTable 8. Association between primary GRS (obtained from the 23andMe replication study) for cataracts or myopia, and incident dementia, AD, and VaD among participants older than 60 at baseline. Models adjusted for age, sex, and first 10 principal components.

Exposure	Outcome	n	Adjusted OR (95% CI)
Cataracts-GRS	All-cause dementia	150,858	1.02 (0.99-1.05)
Cataracts-GRS	AD	150,858	1.00 (0.96-1.05)
Cataracts-GRS	VaD	150,858	**1.09 (1.03-1.16)**
Myopia-GRS[a]	All-cause dementia	150,765	1.00 (0.97-1.03)
Myopia-GRS[a]	AD	150,765	1.01 (0.97-1.06)
Myopia-GRS[a]	VaD	150,765	1.01 (0.95-1.07)

[a]Additionally adjusted for glasses use.

Abbreviations: GRS = genetic risk score; AD = Alzheimer disease, VaD = vascular dementia

© 2024 Ferguson EL et al. *JAMA Network Open.*

eTable 9. Association between secondary GRS (obtained from GERA cohort) and incident dementia, AD, and VaD among participants older than 60 at baseline. Models adjusted for age, sex, and first 10 principal components.

Exposure	Outcome	n	Adjusted OR (95% CI)
Cataracts-GRS	All-cause dementia	183,439	**1.23 (1.20-1.26)**
Cataracts-GRS	AD	183,439	**1.30 (1.25-1.35)**
Cataracts-GRS	VaD	183,439	**1.20 (1.14-1.26)**
Myopia-GRS[a]	All-cause dementia	183,320	1.01 (0.99-1.04)
Myopia-GRS[a]	AD	183,320	1.03 (0.99-1.07)
Myopia-GRS[a]	VaD	183,320	0.96 (0.91-1.01)

[a]Additionally adjusted for glasses use.

Abbreviations: GRS = genetic risk score; AD = Alzheimer disease, VaD = vascular dementia

© 2024 Ferguson EL et al. *JAMA Network Open.*

eTable 10. Associations between AD-GRS, with and without *APOE*, and vision outcomes. Models adjusted for age, sex, and first 10 principal components.

**Outcome**	**n**	**Adjusted OR (95% CI)**	**Adjusted OR without *APOE* (95% CI)**
Self-Reported Cataracts	183,439	1.00 (0.98-1.02)	0.98 (0.96-1.00)
Self-Reported and ICD Diagnoses of Cataracts	183,439	0.99 (0.98-1.00)	**0.98 (0.97-0.99)**
Myopia	40,687	**0.97 (0.95-0.99)**	0.98 (0.96-1.01)
Binary 20/40 Vision	41,646	0.97 (0.92-1.02)	1.00 (0.95-1.05)

Abbreviations: GRS = genetic risk score; AD = Alzheimer disease, *APOE* = apolipoprotein E (*APOE*) gene

© 2024 Ferguson EL et al. *JAMA Network Open.*

eTable 11. Summary reverse causation MR estimates for: 1) the association between AD and self-reported and *ICD* diagnoses of cataracts, 2) the association between AD and myopia, and 3) the association between AD and binary 20/40 vision. Models adjusted for age, sex, and first 10 principal components to proxy for genetic ancestry.

**MR Estimate**	**OR (95% CI)**
**MR estimate of AD on visual impairment (reverse causality)**	
1. Association between AD and self-reported and ICD diagnoses of cataracts	
IVW	0.99 (0.96-1.01)
MR-Egger	0.99 (0.96-1.03)
Weighted-Median	0.99 (0.97-1.01)
Weighted-Mode	1.00 (0.97-1.01)
2. Association between AD and myopia	
IVW	0.96 (0.92-1.00)
MR-Egger	0.98 (0.93-1.03)
Weighted-Median	0.96 (0.93-0.99)
Weighted-Mode	0.97 (0.94-1.01)
3. Association between AD and binary 20/40 vision	
IVW	0.93 (0.86-1.00)
MR-Egger	0.96 (0.88-1.05)
Weighted-Median	0.95 (0.88-1.03)
Weighted-Mode	0.95 (0.88-1.03)

Abbreviations: AD = Alzheimer disease, MR = Mendelian randomization

© 2024 Ferguson EL et al. *JAMA Network Open.*

eFigure 1. Association between 43 cataracts SNPs and both cataracts and dementia. MR estimates for the effect of cataracts (primary) are shown for inverse variance weighted (IVW), MR-Egger (which adjusts for pleiotropy), weighted-median, and weighted-mode.

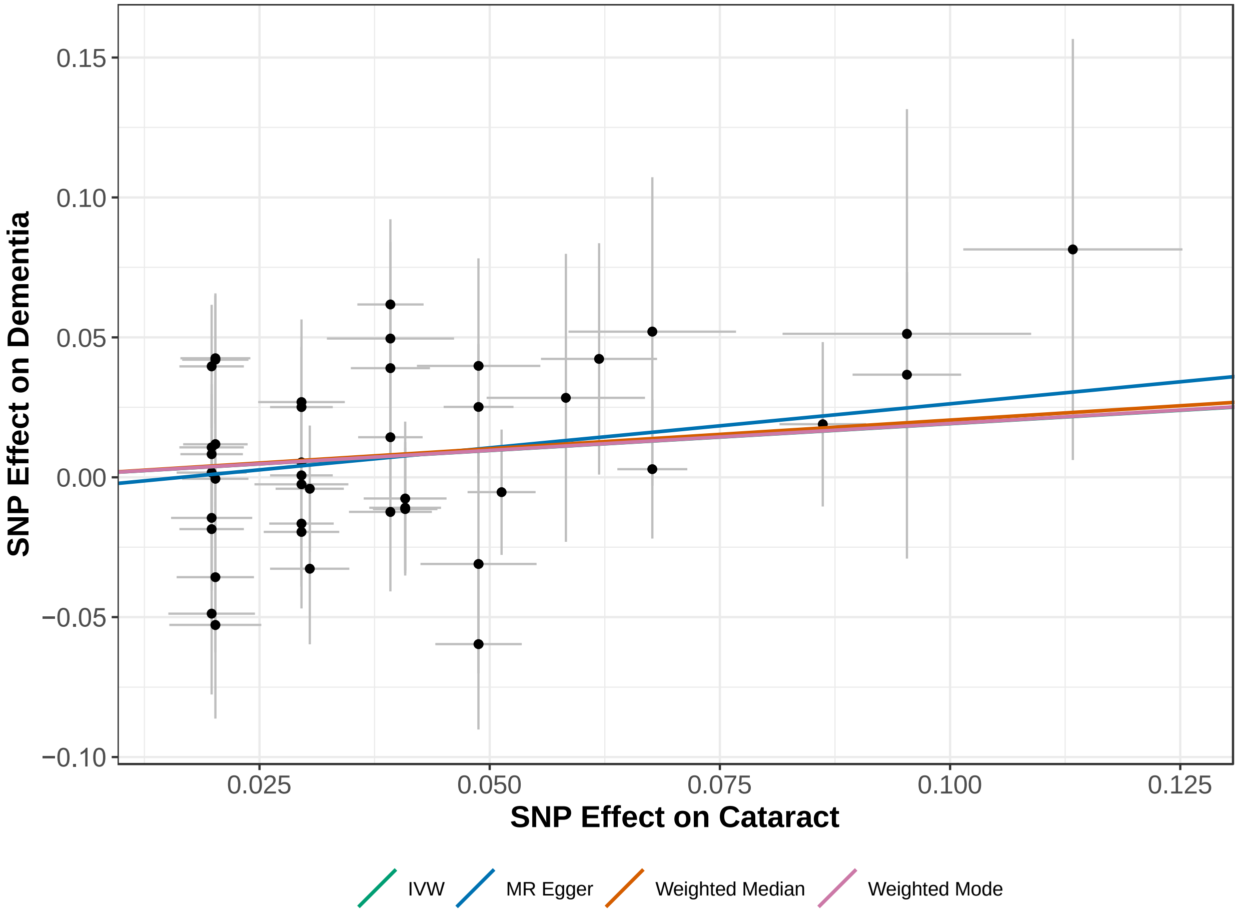

© 2024 Ferguson EL et al. *JAMA Network Open.*

eFigure 2. Association between 43 cataracts SNPs and both cataracts and vascular dementia. MR estimates for the effect of cataracts (primary) are shown for inverse variance weighted (IVW), MR-Egger (which adjusts for pleiotropy), weighted-median, and weighted-mode.

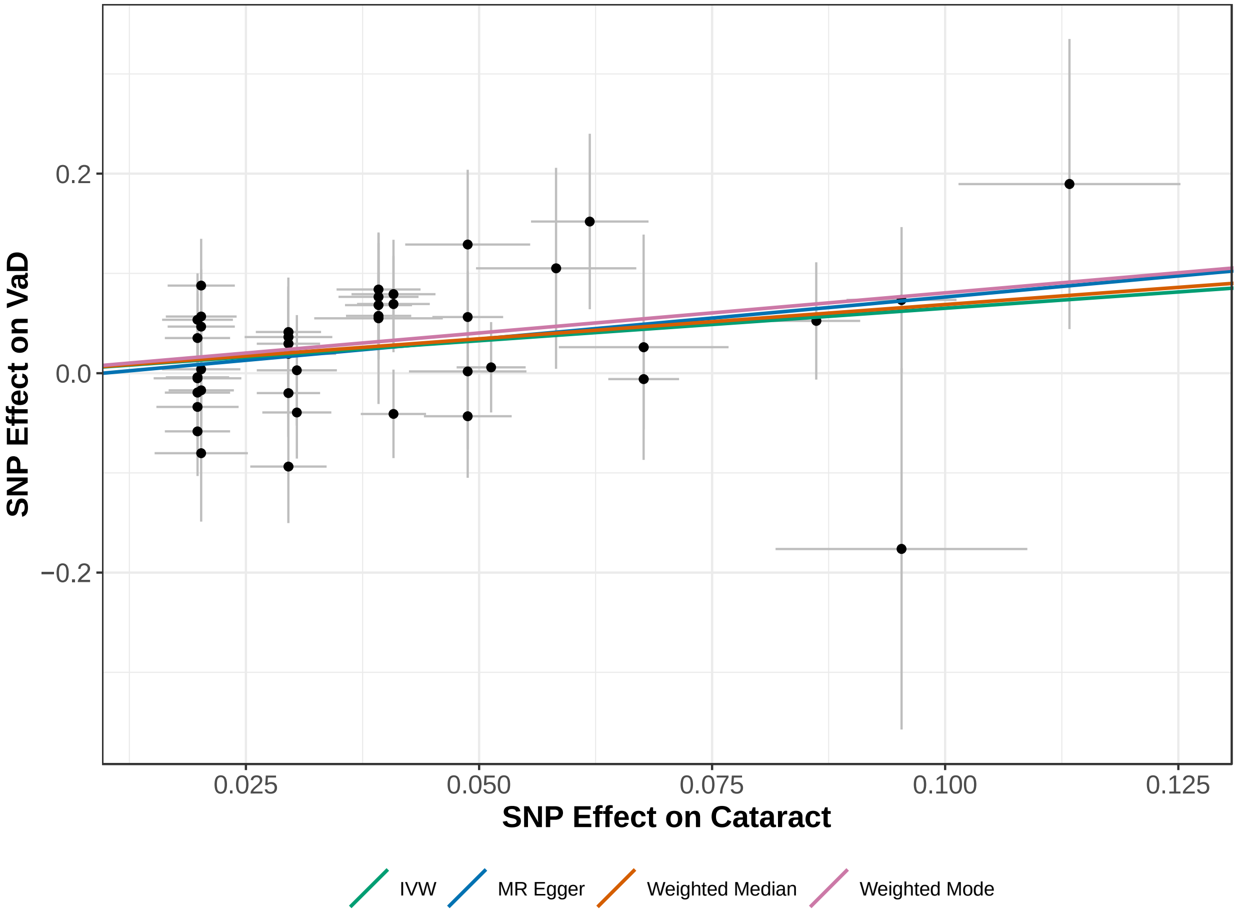

© 2024 Ferguson EL et al. *JAMA Network Open.*

eFigure 3. Association between 27 AD SNPs and both cataracts and dementia. MR estimates for the effect of AD on cataract are shown for inverse variance weighted (IVW), MR-Egger (which adjusts for pleiotropy), weighted-median, and weighted-mode.

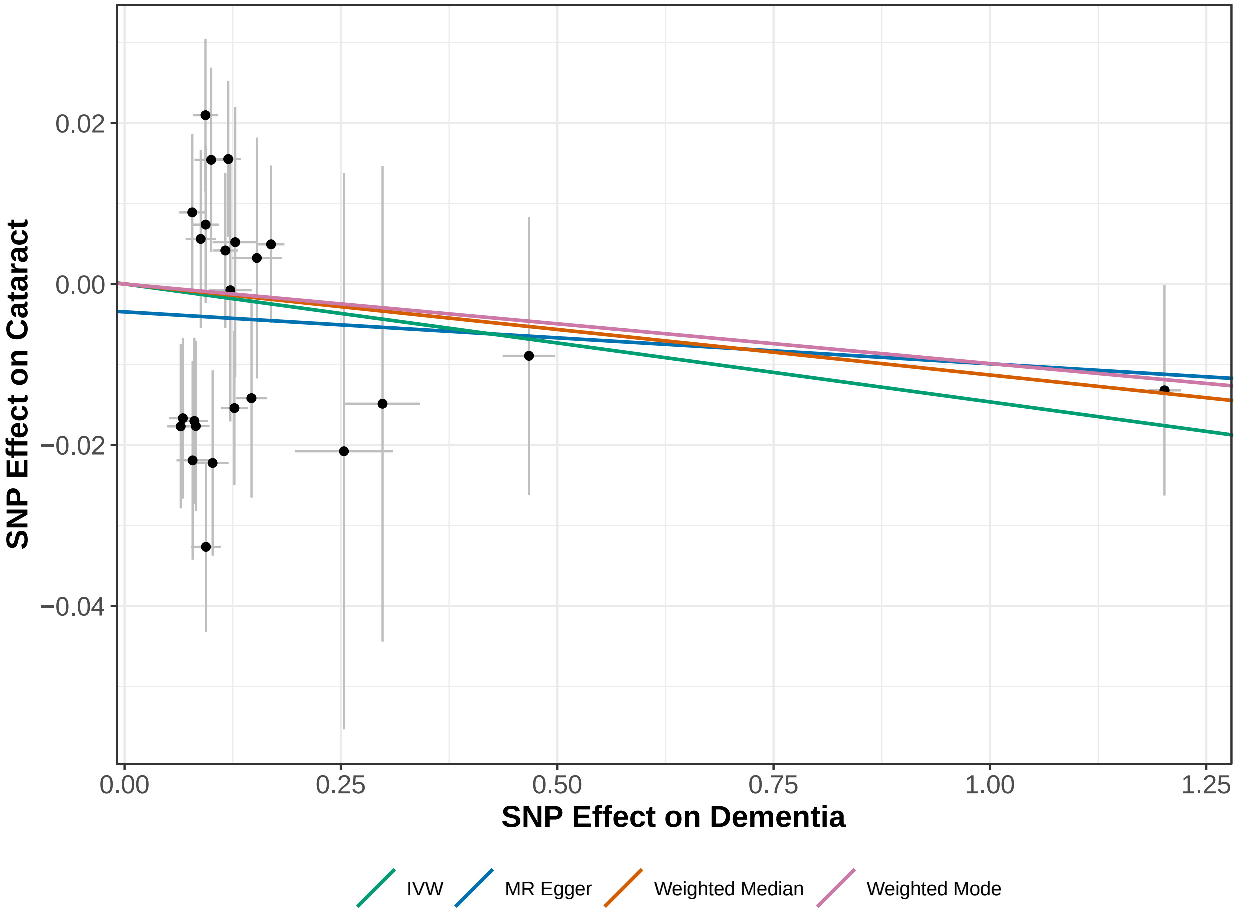

© 2024 Ferguson EL et al. *JAMA Network Open.*
