# Supplementary material for: Visual Impairment, Eye Conditions, and Diagnoses of Neurodegeneration and Dementia
Source: JAMA Netw Open. 2024 Jul 30;7(7):e2424539. doi: 10.1001/jamanetworkopen.2024.24539 (PMC11289698; doi:10.1001/jamanetworkopen.2024.24539)
Supplement: Supplement 2. — Data Sharing Statement [file jamanetwopen-e2424539-s002.pdf]

Data Sharing Statement

Ferguson. Visual Impairment, Eye Conditions, and Diagnoses of Neurodegeneration and Dementia. *JAMA Netw Open*. Published July 30, 2024.
doi:10.1001/jamanetworkopen.2024.24539

Data
Data available: No

Additional Information
Explanation for why data not available: Researchers can apply to access the data used in this study from UK Biobank (http://www.ukbiobank.ac.uk/).
